# Child Tax Credit, Spending Patterns, and Mental Health: Mediation Analyses of Data from the U.S. Census Bureau’s Household Pulse Survey during COVID-19

**DOI:** 10.3390/ijerph20054425

**Published:** 2023-03-01

**Authors:** JungHo Park, Sujin Kim

**Affiliations:** 1 Department of Housing & Interior Design (AgeTech-Service Convergence Major), College of Human Ecology, Kyung Hee University, Seoul 02447, Republic of Korea; 2 Department of Environmental Planning, Graduate School of Environmental Studies, Seoul National University, Seoul 08826, Republic of Korea; 3 Division of Economy and Society, The Seoul Institute, Seoul 06756, Republic of Korea

**Keywords:** generalized anxiety disorder (GAD), major depressive disorder (MDD), child tax credit (CTC), spending pattern, mediation analysis, household pulse survey (HPS)

## Abstract

This study examined the relationship between the receipt of COVID-19 child tax credit and adult mental health problems in the United States, and we explored whether and the extent to which a wide range of spending patterns of the credit—15 patterns regarding basic necessities, child education, and household expenditure—mediated the relationship. We used COVID-19-specialized data from the U.S. Census Bureau’s Household Pulse Survey, a representative population sample (N = 98,026) of adult respondents (18 and older) who participated between 21 July 2021 and 11 July 2022. By conducting mediation analyses with logistic regression, we found relationships between the credit and lower levels of anxiety (odds ratio [OR] = 0.914; 95% confidence interval [CI] = 0.879, 0.952). The OR was substantially mediated by spending on basic necessities such as food and housing costs (proportion mediated = 46% and 44%, respectively). The mediating role was relatively moderate in the case of spending on child education and household expenditure. We also found that spending the credit on savings or investments reduces the effect of the child tax credit on anxiety (−40%) while donations or giving to family were not a significant mediator. Findings on depression were consistent with anxiety. The child tax credit–depression relationships were substantially mediated by spending on food and housing (proportion mediated = 53% and 70%). These mediation analyses suggested that different patterns of credit spending are important mediators of the relationship between the receipt of the child tax credit and mental illnesses. Public health approaches to improve adult mental health during and after the COVID-19 pandemic need to consider the notable mediating role of spending patterns.

## 1. Introduction

As the Coronavirus Disease 2019 (COVID-19) pandemic enters its fourth year, many countries around the globe are in a better place with dealing with the disease but still face a crisis in relation to people’s mental health [1,2,3,4]. The pandemic has increased the risks related to poor mental health—stress and fear, loss of income, job insecurity, etc.—whereas defensive aspects—outdoor activities, socialization, educational opportunities, accessibility to health care services, etc.—worsened markedly [5,6]. The accumulated scale of mental health problems is so enormous that it calls for comprehensive and all-encompassing societal support across the globe [6]. The Global Burden of Diseases, Injuries, and Risk Factors (GBD) 2020 study aligned by the World Health Organization (WHO) estimated that, during the COVID-19 pandemic, symptoms of depression and anxiety were the most prevalent mental disorders and they increased by 27.6% and 25.6%, respectively, across the world [7].

In the United States, in response to the global health crisis, a wide range of economic and social safety net programs have been newly developed (e.g., stimulus check) and expanded (e.g., child tax credit, unemployment insurance). Daily conflicts between work and life domains among numerous American households, especially for those with kids, can be eased by programs and policies that recognize a variety of circumstances in which normal households find themselves [5,8,9,10]. Recent studies have reported that socioeconomic programs and policies have proved successful in helping to directly or indirectly improve the mental health of beneficiaries [11,12]. Beyond the general understanding of the link, however, the underlying mechanisms linking government support to mental health have yet to be examined.

### 1.1. Expansion of the U.S. Child Tax Credit Amid the Crisis of Mental Health during COVID-19

Considering the enormous socioeconomic difficulties among American households resulting from the COVID-19 pandemic, the American Rescue Plan Act of 2021 made critical expansions to the child tax credit (hereafter, CTC). CTC was first introduced by The Taxpayer Relief Act of 1997 in order to ease the economic burden on households with children. CTC is a refundable tax credit for dependent children. The tax amount of households with federal income tax to be paid is deducted, and cash benefits are also paid from the federal government.

An important element of the American Rescue Plan Act included an expansion of CTC with advance payments starting from July 2021, a “child allowance” which was expected to sharply elevate the level of child poverty [13]. The amount of CTC was increased from USD 2000 to USD 3600 for eligible children (aged 5 or younger) and USD 3000 for other eligible children (aged 17 or younger). To provide much-needed economic relief, the American Rescue Plan Act revised the credit to be entirely refundable and provided on a monthly basis for the initial six months in contrast with the previous rate of once per year. By the middle of July 2022, 88% of households with children received payments of USD 250 (or USD 300) per child every month. The changes included expanding the qualification to age 17, making the CTC entirely refundable, raising the credit for wide lower- and middle-income taxpayers, with greater increases for younger kids, and disbursing half of these payments every month rather than after a household files their taxes, beginning in July 2021 [14].

As is the case with CTC in the U.S., many countries across the world expanded their child allowance (interchangeably child benefit, [15,16]) during and even before the COVID-19 pandemic. This included child benefit in the U.K., Canadian child benefits in Canada, child tax benefit in South Korea, and child allowance in Japan, among many others. A growing body of COVID-19 studies in the U.S. has also found that there are relationships between income supplements and mental health, such as stimulus checks [17], economic impact payment [18], unemployment insurance [19], supplemental nutrition assistance programs [20], and temporary assistance for needy families [21,22], among many others. Focusing on child mental health, studies identified the positive role of cash assistance. For example, at ages 25 and 30, participants from members of an American Indian tribe whose households received cash payments experienced less anxiety and depression, indicating that cashable payments to households may result in longer-term benefits for children’s mental wellbeing [23].

Yet, there is restricted knowledge of the fundamental processes between the receipt of CTC and adult mental health. On the one hand, the cash benefit may directly improve mental health simply because of the receipt of the credit. During the pandemic, policies that have provided greater financial assistance or reduced the risk of financial insecurity have also been shown to provide opportunities for good health or to directly improve health [22]. On the other hand, there may be indirect ways through which the receipt of CTC may improve mental health. CTC may also increase the perceived manageability of debt while easing mental health stress [24].

### 1.2. Diverse Spending Patterns of the Child Tax Credit

Maslow’s hierarchy of needs may explain the human motivation behind consumer behaviors and spending patterns, particularly in urgent situations such as the COVID-19 pandemic [25,26,27]. The theory describes five hierarchical levels—physiology, safety, love and belonging needs, esteem, and self-actualization—which may be related to a wide range of spending patterns of cashable CTC in the COVID-19 pandemic. Moreover, the COVID-19 crisis increased consumer fear and uncertainty in relation to spending decisions due to the loss of income and fears of contagion [26]. Therefore, consumers may focus more on satisfying basic needs than on fulfilling higher levels of needs [28].

Considerable evidence supports the primary uses of CTC for basic needs [29]. CTC allowed households to cover daily expenses, including housing cost, more and higher-quality food, clothing, and other necessities for their children [30,31]. The expanded monthly payments might help support adults in making ends meet when pandemic-induced inflation raises the prices of essential items [32]. Especially for the poorest parents with children, the added monthly income from the credit helps them secure urgent daily items [32].

In particular, previous literature found that missing routine meals due to economic issues is among the most serious hardships related to mental health problems [10]. CTC significantly lowered qualified households’ food insecurity and helped them afford balanced and healthy foods for their kids and pregnant women [33]. Since the distribution of CTC, food security has improved dramatically for all racial/ethnic subgroups, but particularly for Black and Latino people [34]. Although adult members of households with kids are more likely to suffer a lack of food, the households experienced a three percentage point reduction between the surveys performed before and after the credit payments [35]. Food insecurity is related to mental illnesses due to the consequent fear, depression, anxiety, stigma, and stress [36]. In case of Canada, lack of food was independently related to poor mental health in the early days of the COVID-19 pandemic [37].

As for expenses relating to child education during the pandemic, parents have faced unprecedented hardships due to limited transportation from and to school, isolation measures, and the closure of childcare centers and schools [38]. CTC recipients may spend the payments on educational materials and class activities for their kids. A recent study reported that the credit payments resulted in a significant difference in parents’ capacity to pay off basic school items and class activities for their kids, benefiting children’s health and educational opportunities [32]. Pre-pandemic studies also found that kids in households who benefited from income support showed better mental health in adulthood. This implies a positive and long-term benefit of CTC, which will be realized in the future [39,40].

In the case of household expenditure, CTC effectively and efficiently lowered financial burdens for qualified households, as demonstrated by their reduced credit card debt and lower risk of eviction. The significant expansions have enormously lowered child poverty, lifting an added 4.1 million kids above the nationwide poverty level by 40% [41]. CTC also helped households save for emergencies, pay off debt [30], and work additional hours outside the home [32]. During the prevention and control of COVID-19, social support could help reduce a variety of symptoms of mental illnesses [42]. Additionally, a greater amount of debts were associated with a higher level of stress and, in turn, worse mental health [43].

These different spending patterns of CTC may be mediators in the relationship between the receipt of CTC and mental health during the pandemic. The National Child Tax Credit Survey showed that the credit payments have successfully lowered economic stress, with 70% of survey participants stating that the payments made them much less stressed about household expenditure [41]. In contrast, a study found a series of mixed results regarding the effects of CTC on adult depression and parental stress [30].

Given the historical expansion of CTC and its substantial effects on American households, it is important to examine the relationships between the CTC monthly payments and mental health outcomes in the pandemic. Parental mental health plays a central role in securing the psychological well-being of the entire household, reducing parenting irritability, parental burnout, and verbal conflict between couples [44,45,46,47,48]. Furthermore, studies have revealed that deterioration of parental mental health occurs due to not only social distancing and closures [49,50,51] but also extended time spent on childcare and homeschooling [5]. Understanding the effects of public assistance, including CTC, on adult mental well-being is also critical for informing public health policies that better resolve mental health needs related to urgency and enhance the psychological well-being of adult parents and their household members.

Few population-based studies have focused on whether and the extent to which a wide range of spending patterns of the CTC monthly payments may mediate the relationship between CTC and mental health in the pandemic. Population-based research is critical since clinical study cases may not represent the entire population. Moreover, a wide range of daily spending patterns may be critical to draw health implications for the entire population.

We aim to contribute to an expanding body of literature that shows the importance of social policies on mental health, particularly during the pandemic [22]. This study used population-based and pooled cross-sectional datasets to analyze the relationship between the receipt of CTC and adult mental health problems. We also explored whether and the extent to which fifteen different patterns of the credit usage mediated the relationship between the receipt of the credit and mental health outcomes.

**Figure 1:**
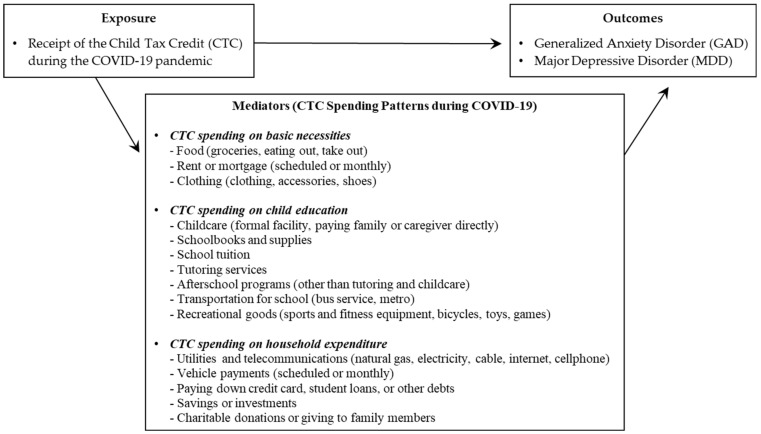
Conceptual model of the relationship between the receipt of the COVID-19 child tax credit (exposure), mental health problems (outcomes), and spending patterns of the credit (mediators) in the COVID-19 pandemic.

Figure 1 shows an overall conceptual framework of our study that attempts to link CTC (exposure) and mental health problems (outcome) with salient mediators of 15 different spending patterns of CTC. The following section describes the Household Pulse Survey data—primary data of the study—and variable definitions, along with the specification of mediation analysis models.

## 2. Materials and Methods

### 2.1. U.S. Census Bureau’s HPS Data during COVID-19

The Household Pulse Survey (HPS) is a nationally representative survey deployed by the U.S. Census Bureau and the U.S. National Center for Health Statistics (NCHS), as well as other federal organizations. It surveys the socioeconomic and health impacts of the pandemic on adult households in the U.S. HPS was conducted biweekly (or weekly in early periods of the pandemic) and largely consists of three phases starting from 23 April 2020: phase 1 (23 April–21 July 2020, survey weeks 1 to 12), phase 2 (19 August–26 October 2020, survey weeks 13 to 17), and phase 3 and following subphases (28 October 2020–ongoing, survey weeks 18 and later). We utilized a one-year portion of HPS (21 July 2021–11 July 2022, survey weeks 34 to 47) when new survey questions about CTC were introduced and became available for analysis. Note that we did not use data collected later than survey week 47 because CTC-related questions were not asked anymore. This study utilized the Public Use File (PUF) of HPS—microdata which are free to download—which provides survey answers from individual respondents (see Supplementary Table S1 for details about the sample size of PUF microdata by survey week and phase).

### 2.2. Study Variables

#### 2.2.1. Outcome: Mental Health Problems

Two types of self-reported measures—Generalized Anxiety Disorder (GAD; GAD-2 [52]) and Major Depressive Disorder (MDD; PHQ-2 [53])—were adopted to identify the level of mental health of CTC recipients. The two questions measure the frequencies of the symptoms of anxiety and depression in the past week. The base question of GAD-2 and PHQ-2 is “in the past week, how often have you been distracted by any of the following difficulties?” The two subitems of GAD-2 are “feeling nervous, anxious or on edge” and “cannot stop or control worrying” while the items for PHQ-2 are “having little interest or pleasure in doing things” and “feeling down, depressed, or hopeless.” The responses from survey participants were coded by integers, such as not at all = 0, several days = 1, more than half the days = 2, and nearly every day = 3. Values for each item were summed and then categorized into binomial outcomes, such as four or higher points from GAD-2 as GAD and from PHQ-2 as MDD. The thresholds of PHQ-2 and GAD-2 have been validated for diagnosed GAD and MDD [52,53] (see Supplementary Table S2 for more information regarding survey questions and answers).

#### 2.2.2. Exposure: Receipt of the Child Tax Credit

We specified a binary exposure variable using the following survey question: “In the last 4 weeks, did you receive a refund from your 2021 tax return?” with answer options of yes (=1) or no (=0). To narrow down the sample of our analysis, we dropped respondents who did not respond to another CTC-related question ‘Considering your spending of the CTC monthly payments, did you: (a) mostly spend it, (b) mostly save it, or (c) mostly use it to pay off debt’.

#### 2.2.3. Mediator: Spending Patterns of the Child Tax Credit

We measured the spending patterns of CTC on the basis of a multiple-choice survey question: “What did you and your household mostly spend the “Child Tax Credit” portion of your refund on? Select all that apply”. Answer options of yes or no were available for the 15 different patterns of CTC spending, which were grouped into three types: CTC spending on basic necessities (food, rent or mortgage, and clothing), CTC spending on child education (childcare, schoolbooks and supplies, school tuition, tutoring services, afterschool programs, transportation for school, and recreational goods), and CTC spending on household expenditure (utilities and telecommunications, vehicle payments, paying off credit cards or debts, savings or investments, and donations or giving money to family). We ran the same mediation analysis 15 times using one mediating variable at a time to avoid multicollinearity between the mediators (see Pearson correlation coefficients between −0.001 and 0.3925 as shown in Supplementary Table S3).

#### 2.2.4. Covariate: Characteristics of Survey Participants

We considered individual and household characteristics ranging from demographic attributes to social and economic statuses (SES), health insurance status, and location of residence. Demographic characteristics consisted of age, sex, race and ethnicity, marital status, count of kids, and number of household members. SES included education and household income. To control for health-related covariates, we considered the status of public and private health insurance. In addition, two sets of geographic identifiers were included in the model to control for the location of residence of survey respondents, such as 50 states and the Washington, D.C., and 15 largest metropolitan statistical areas (MSAs). These geographic variables were included to reflect differences in the mental health outcomes between distinct areas across the nation.

Table 1 shows descriptive statistics of variables of the entire cases, as well as separately for the diagnosis of anxiety and depression. The overall effect of the pandemic on mental health was not distributed equally across the population subgroups, which is in line with previous literature on relationships between public support and mental health [19,20,21,22,32,34,35,54].

### 2.3. Model Specification

We adopted Stata MP version 13.1 program (StataCorp, College Station, TX, USA) across analysis models We used logistic regression models for our mediation analysis. We used mediation analyses (*paramed* in Stata program; [55]) which were developed by VanderWeele [56]. The method allows researchers to decompose a total effect into direct and indirect effects on the basis of counterfactual framework. VanderWeele’s method can also address limitations of the traditional approach developed [57], which omits potential interrelationships between exposure variables and mediating variables. By using 500 bootstrapping resamples and producing 95% bias-corrected confidence intervals, we estimated direct and indirect effects in the models. Additionally, we estimated how much of the total effect is mediated in the model using the following equation: odds ratio [indirect effect]/odds ratio [total effect] × 100%.

## 3. Results

### 3.1. Spending Patterns of CTC and Anxiety

The first model result (CTC spent on food) at the top of Table 2 shows a negative relationship between the receipt of CTC and GAD (odds ratio [OR] of total effect = 0.914; 95% confidence interval [CI] = 0.879, 0.952) after controlling for all covariates. The association between CTC and GAD was significantly mediated by the spending pattern of using CTC to purchase food by 46% (OR of indirect effect = 0.958; 95% CI = 0.938, 0.980). Additionally, CTC spending on housing costs (rent or mortgage) substantially mediated the association between CTC and GAD by 44%. In contrast, using CTC to buy clothing did not significantly mediate the association between CTC and GAD.

As for CTC spending related to child education, we find that the association between CTC and GAD is significantly mediated when the recipients use the credit to pay for childcare (OR of indirect effect = 0.988; 95% CI = 0.981, 0.995, proportion mediated = 13%), school tuition (OR of indirect effect = 0.994; 95% CI = 0.989, 0.999, proportion mediated = 7%), and transportation costs of traveling to and from school (OR of indirect effect = 0.988; 95% CI = 0.981, 0.995, proportion mediated = 13%). Other spendings on child education—schoolbooks and supplies, tutoring services, afterschool programs, and recreational goods, etc.—are insignificant or weakly significant mediators in the association between CTC and GAD.

Most CTC spending on household expenditure emerges as a significant mediator in the relationship between CTC and GAD. Using CTC to pay for utilities and telecommunications significantly mediated the relationship between CTC and GAD by 36%. Similarly, CTC spending on vehicle payments and credit cards or debts mediated the relation between CTC and GAD by 13% and 27%, respectively. Unlike all the other mediators in this article, the use of CTC on savings or future investments appears to be associated with a higher level of GAD (OR of indirect effect = 1.043; 95% CI = 1.026, 1.060, proportion mediated = −40%). Spending CTC on charitable donations or giving to family did not have a significant mediating role in the model.

### 3.2. Spending Patterns of CTC and Depression

Table 3 includes mediation analysis results with MDD as the outcome variable, which is mostly consistent with the model result with GAD. When using CTC spending on basic necessities as a mediator, we find negative associations between CTC and MDD across models with different mediators. The relationships between CTC and MDD were significantly and substantially mediated by CTC spending on food (proportion mediated = 53%) and housing costs (proportion mediated = 70%). Notably, more than half of the total effects of CTC on MDD were mediated by those spending patterns. CTC spending on clothing was not a significant mediator.

Among CTC spending on child education, we find a significant mediating role only in the cases of childcare spending (OR of indirect effect = 0.989; 95% CI = 0.981, 0.997, proportion mediated = 13%) and cost of transportation to and from school (OR of indirect effect = 0.997; 95% CI = 0.995, 1.000, proportion mediated = 4%). The other spendings on child education were not a significant mediator in the model; these included schoolbooks and supplies, school tuition, tutoring services, afterschool programs, and recreational goods.

Turning to CTC spending on household expenditure, we discovered significant mediating roles when the credit was spent on utilities and telecommunications (OR of indirect effect = 0.968; 95% CI = 0.957, 0.980, proportion mediated = 46%), vehicle payments (OR of indirect effect = 0.990; 95% CI = 0.984, 0.997, proportion mediated = 13%), and paying off credit cards or debts (OR of indirect effect = 0.976; 95% CI = 0.963, 0.989, proportion mediated = 29%). As was the case for the GAD model, CTC spending on savings or investments was associated with a higher level of MDD (OR of indirect effect = 1.029; 95% CI = 1.008, 1.051, proportion mediated = −33%). Spending CTC on charitable donations or giving money to family was not a significant mediator in the model.

## 4. Discussion

### 4.1. Key Findings of Mediation Analyses

Overall findings indicate that CTC recipients in the COVID-19 pandemic are at notably decreased risk of anxiety and depression and that a substantial proportion of this lowered risk stems from spending patterns of the credit on basic necessities, child education, and household expenditure. These findings correspond with previous results about a wide range of spending patterns of CTC and lowered level of mental illnesses among CTC recipients [24,30,31,32,34], suggesting that the risk of mental health problems is lowered for CTC recipients who spent the monthly credit on life essentials during the pandemic.

Our findings show significant associations of the receipt of CTC with anxiety and depression, in addition to the important mediating roles of spending patterns of the credit in the U.S. in the pandemic. The findings are consistent with early COVID-19 studies in that CTC plays a positive role in alleviating mental illnesses during the pandemic. Very few studies have explored whether a variety of spending patterns of CTC might be mediators of the relationship between the receipt of the credit and mental health outcomes. Furthermore, we have limited knowledge about the extent to which these relationships are mediated by the spending pattern of the credit. We found that some specific spending patterns were partial but significant mediators of the association between the receipt of CTC and mental health outcomes, while others were not.

We made three key discoveries about the relationships between the receipt of CTC, spending patterns, and mental health outcomes. First, we found the strongest mediators were spendings on food and housing as people’s most fundamental needs. In particular, the expanded CTC helps low- and moderate-income households to decrease their financial stresses as it allows them to buy daily necessities, increasing opportunities for children’s education [32]. Additionally, permanent payment of expanded benefits may reduce food insecurity during the pandemic situation [34] and help recipients secure stable housing [58]. The need for continued expansion of CTC in terms of mental health improvement is also found in other government programs such as EITC [59]. After expiration of CTC monthly payments, food insufficiency was increased in households with children [60] and they experienced poverty, especially in households including Black and Latinx children [32].

Second, we found that spending of CTC on childcare and transportation for school mediated the relationship between the receipt of CTC and mental health problems. Parents used the CTC to buy toys and engage in activities with their children; thus, the bond between parents and children improved. Additionally, parents purchased more food or high-quality food [41]. A third of spending CTC was school related [35] and educational opportunities increased [32]. In addition, our results showed that use of CTC for household expenditure was a significant mediator of the relationship. The cash form of CTC appears to play a crucial role in securing flexibility and diversity of uses of the credit. In terms of flexibility, a recent study supports the rising importance of flexibly designed mental health measures interventions for both kids and parents [48]. The current policy trend of low-income and middle-income countries is being adapted or expanded as cash transfer programs in order to overcome the pandemic crisis [11]. These cash transfer programs should not only deal with food insecurity but should also focus on addressing long-term mental health disruptions resulting from the ongoing pandemic. A study by Brookings suggests that CTC is an effective tool in terms of cutting child poverty in the short term and will help to increase household social mobility in the long term [61]. More generous cash transfer is a powerful tool, relieving the negative mental, physical, and behavioral health responses to stress created by unemployment and wage loss [22]. Continued and regular cash payment is important, as it alleviates stress [58].

Third, we found spending on savings and investments was related to a higher level of anxiety and depression, which indicates the opposite role against other mediators used in our study. This finding calls for policy makers to consider negative effects of CTC on recipient households and their consumption. We need to consider that the prolonged COVID-19 situation aggravated household’s economic burdens [45]. This is different from the situation in which people received public subsidies in the early stages of COVID-19; at that time, parents spent these subsidies on daily necessities and debts [35]. On the other hand, spending on saving or investment in this study was intended to prepare for financial difficulties due to prolonged COVID-19; thus, saving or investments were not positive acts for mental health. In addition, the expanded CTC may result in low-income earners making less of an effort to find work [62], which will have the opposite effect, leading to a decrease in income and household assets. In this situation, it is judged that savings and investment negatively affect mental health because CTC should be prioritized in food and housing expenditure, and savings or investments can be made later.

### 4.2. Limitations and Future Research

Our findings need to be considered in light of four limitations. First, the HPS did not include previous and chronic indicators of the survey participants’ mental health, and therefore we were not able to control for pre-pandemic existence of a diagnosis of mental illness. Second, as our study did not analyze the initial pandemic situation when CTC was not expanded, we could not compare how much the mental health of parents improved in response to CTC. Third, we did not consider temptation goods such as cigarettes or alcohol as a food purchasing item in the HPS checklist, as food purchasing only included groceries, eating out, and take out. Thus, we could not find whether temptation goods affected the improvement of mental health [63]. Fourth, we did not compare the characteristics of consumption patterns according to differences in household income and other socioeconomic characteristics which may interact with spending patterns of CTC. A number of studies found that low-income families became more vulnerable to food insecurity and economic problems during the pandemic [13,33,34,54]. Further studies may focus on lower-income families who are likely to continue and even expand spending CTC on essential items (e.g., food, rent, utility bills, children’s education) than on savings and investment as the pandemic enters its fourth year.

### 4.3. Policy Implications

We can provide three policy implications to help improve mental health among CTC recipients by considering the important mediating role of their spending patterns during the COVID-19 pandemic. First, the government should consider whether the expanded CTC should be made permanent to promote households’ health and reduce health differences between income groups [22]. Considering the limitation of the government budget, subsidies may result in financial burden in the long run. An unexpected discontinuity of CTC—particularly its expanded benefits—may disrupt the spending power of eligible households with children because they are likely to plan their spending on the basis of CTC benefits. In the midst of working toward a permanent expansion of CTC, community partners, lawmakers, and federal officials need to secure the continuity of CTC with regard to spending patterns [32].

Second, policy makers should design public financial assistance to be spent on buying healthy food and well-being products to improve recipients’ mental health. Previous studies showed that stress made consumers buy things on impulse [63,64,65]. In response to a historical crisis, some people even tend to buy hedonic and harmful products within a few weeks, as well as spending money on temptation goods [28]. These spending behaviors may not be sustainable and desirable in the long term, despite their temporary effect on stress. Especially in a pandemic, careful choice of healthy foods is important for the population’s health [66]. During the COVID-19 pandemic, studies found that some consumers preferred purchasing healthy foods to buying other non-healthy goods [67]. Thus, health policy makers should consider ways to promote the purchase of health-friendly goods, beyond simply increasing the number and size of public benefits.

Third, policy providers may suggest appropriate timing and duration of the assistance provided to population subgroups. We found that spending patterns (e.g., foods, children education, saving) had different effects on improving recipients’ mental health. Spending on foods and child education affected mental health positively, while spending on saving and debt had a negative effect on mental health. This was probably caused by differences in household income and other socioeconomic characteristics. Recent studies found disproportionate difficulties of daily spending among socioeconomically disadvantaged households [68,69].

## 5. Conclusions

We conducted a population-based study on the relationship between the receipt of CTC and mental health outcomes during the COVID-19 pandemic, with a special focus on the mediating roles of spending patterns of credit in the U.S. Key results support the hypothesis that the receipt of CTC is related to lower levels of anxiety and depression, and these relationships were partially mediated by spending patterns of the credit during the pandemic. Public health approaches to improve population mental health during and after the pandemic need to consider the important mediating roles of spending patterns of CTC.

## Figures and Tables

**Table 1:** Descriptive statistics of exposure, mediators, and covariates.

Variables	Full Sample (n = 98,026), %, Mean (SD)	Generalized Anxiety Disorder		Major Depressive Disorder	
		Yes (n = 30,604), %, Mean (SD)	No (n = 67,422), %, Mean (SD)	Yes (n = 20,367), %, Mean (SD)	No (n = 77,659), %, Mean (SD)
Exposures, mean (SD)					
Receipt of CTC during the COVID-19 pandemic	0.169 (0.375)	0.173 (0.378)	0.167 (0.373)	0.174 (0.379)	0.167 (0.373)
Mediators, mean (SD)					
CTC spending on basic necessities as mediator					
CTC spent on food	0.514 (0.5)	0.566 (0.496)	0.488 (0.5)	0.579 (0.494)	0.493 (0.5)
CTC spent on rent or mortgage	0.287 (0.453)	0.364 (0.481)	0.25 (0.433)	0.392 (0.489)	0.255 (0.436)
CTC spent on clothing	0.292 (0.455)	0.321 (0.467)	0.278 (0.448)	0.328 (0.47)	0.281 (0.45)
CTC spending on child education as mediator					
CTC spent on childcare	0.11 (0.313)	0.126 (0.332)	0.103 (0.304)	0.119 (0.324)	0.108 (0.31)
CTC spent on schoolbooks and supplies	0.169 (0.375)	0.197 (0.398)	0.156 (0.363)	0.201 (0.401)	0.16 (0.366)
CTC spent on school tuition	0.05 (0.217)	0.048 (0.214)	0.05 (0.218)	0.048 (0.213)	0.05 (0.218)
CTC spent on tutoring services	0.013 (0.11)	0.015 (0.12)	0.012 (0.105)	0.015 (0.121)	0.012 (0.107)
CTC spent on afterschool programs	0.036 (0.185)	0.037 (0.187)	0.035 (0.183)	0.037 (0.187)	0.035 (0.184)
CTC spent on transportation for school	0.041 (0.197)	0.055 (0.228)	0.033 (0.179)	0.059 (0.236)	0.035 (0.183)
CTC spent on recreational goods	0.043 (0.202)	0.047 (0.211)	0.041 (0.198)	0.044 (0.205)	0.043 (0.202)
CTC spending on household expenditure as mediator					
CTC spent on utilities and telecommunications	0.313 (0.464)	0.403 (0.491)	0.27 (0.444)	0.434 (0.496)	0.276 (0.447)
CTC spent on vehicle payments	0.134 (0.341)	0.181 (0.385)	0.111 (0.314)	0.189 (0.391)	0.117 (0.322)
CTC spent on paying off credit cards or debts	0.186 (0.389)	0.218 (0.413)	0.17 (0.376)	0.218 (0.413)	0.176 (0.381)
CTC spent on savings or investments	0.162 (0.368)	0.104 (0.306)	0.19 (0.392)	0.097 (0.296)	0.182 (0.386)
CTC spent on donations or giving to family	0.012 (0.107)	0.012 (0.107)	0.012 (0.108)	0.011 (0.1)	0.012 (0.109)
Covariates, % of sample					
Demographic characteristics					
Age					
18–24 (Ref)	3.0	4.0	2.5	4.5	2.6
25–34	23.4	27.4	21.4	28.4	21.8
35–44	40.1	40.5	40.0	38.8	40.5
45–54	22.9	19.7	24.5	19.4	24.0
55–64	7.2	5.9	7.8	6.4	7.5
65–74	2.8	2.0	3.1	1.9	3.0
75+	0.6	0.4	0.7	0.5	0.6
Gender					
Female (Ref)	57.1	64.8	53.4	62.1	55.6
Male	42.9	35.2	46.6	37.9	44.4
Race/ethnicity					
Non-Hispanic White (Ref)	56.2	57.5	55.6	54.9	56.6
Non-Hispanic Black	13.3	13.6	13.1	14.4	12.9
Non-Hispanic A and PI	5.6	3.4	6.7	3.8	6.2
Non-Hispanic other	4.2	4.9	3.8	5.4	3.8
Hispanic	20.7	20.6	20.8	21.6	20.5
Marital status					
Unmarried (Ref)	30.0	38.4	26.0	42.4	26.2
Married	70.0	61.7	74.0	57.6	73.8
Children in household					
No child (Ref)	3.9	3.9	4.0	4.7	3.7
One or more children	96.1	96.2	96.0	95.3	96.3
Household size					
Single person (Ref)	0.4	0.4	0.3	0.6	0.3
2-person	3.5	4.0	3.3	4.5	3.2
3-person	21.4	21.9	21.1	21.7	21.3
4-person	33.8	32.4	34.4	30.7	34.7
5-person	21.1	20.3	21.5	20.3	21.3
6 or more persons	19.9	20.9	19.4	22.3	19.2
Socioeconomic status (SES)					
Education					
Less than high school (Ref)	8.5	8.8	8.4	9.1	8.4
High school	27.4	29.0	26.6	32.0	26.0
Some college and AA	31.4	35.4	29.4	37.0	29.7
BA+	32.7	26.8	35.6	22.0	36.0
Household income					
Less than USD 25,000 (Ref)	13.9	19.8	11.0	21.7	11.5
USD 25,000–49,999	24.1	28.0	22.2	31.7	21.7
USD 50,000–74,999	17.2	17.9	16.8	17.1	17.2
USD 75,000–99,999	13.5	12.4	14.1	11.3	14.2
USD 100,000–USD 149,999	17.3	12.9	19.4	10.8	19.3
USD 150,000 and above	14.0	9.1	16.4	7.4	16.1
Health insurance status					
Public health insurance					
No (Ref)	69.7	63.5	72.7	60.9	72.3
Yes	30.3	36.5	27.4	39.1	27.7
Private health insurance					
No (Ref)	25.7	32.8	22.2	36.0	22.6
Yes	74.3	67.2	77.7	64.0	77.4
Location of residence					
15 largest metropolitan statistical area					
None (Ref)	68.8	71.4	67.5	71.0	68.1
New York	4.8	4.1	5.2	3.8	5.1
Los Angeles	3.8	3.2	4.2	3.4	4.0
Chicago	2.6	2.4	2.7	2.4	2.6
Dallas	2.5	2.5	2.6	2.8	2.5
Houston	2.4	2.4	2.4	2.4	2.4
Washington, D.C.	1.8	1.6	2.0	1.6	1.9
Miami	1.6	1.6	1.6	1.5	1.7
Philadelphia	1.8	2.0	1.8	2.0	1.8
Atlanta	1.9	1.7	2.0	1.7	1.9
Phoenix	1.7	1.6	1.8	1.6	1.8
Boston	1.2	1.0	1.4	0.9	1.4
San Francisco	0.9	0.8	1.0	0.8	0.9
Riverside	1.6	1.3	1.7	1.6	1.6
Detroit	1.2	1.2	1.2	1.3	1.1
Seattle	1.2	1.3	1.2	1.2	1.2
50 States and Washington, D.C.	-	-	-	-	-

Note: For simplicity, the descriptive statistics of samples in 50 states and Washington, D.C. were omitted and shown in Supplementary Table S4. Person-level weight was applied in this table. CTC = Child Tax Credit. A and PI = Asian and Pacific Islander. AA = some college or associate degree. BA+ = bachelor’s degree or higher.

**Table 2:** Results from mediation analysis on CTC spending patterns as mediators between CTC receipt and anxiety.

Mediator Variables	CTC Receipt–Anxiety Relationship			
	OR	(95% CI)	*p*	% of Total Effect
CTC spending on basic necessities as mediator				
CTC spent on food				
Direct	0.954	(0.912, 0.998)	0.039	54
Indirect	0.958	(0.938, 0.98)	<0.001	46
Total	0.914	(0.879, 0.952)	<0.001	100
CTC spent on rent or mortgage				
Direct	0.957	(0.919, 0.998)	0.036	56
Indirect	0.965	(0.952, 0.98)	<0.001	44
Total	0.924	(0.888, 0.962)	<0.001	100
CTC spent on clothing				
Direct	0.920	(0.884, 0.959)	<0.001	96
Indirect	0.997	(0.986, 1.009)	0.575	4
Total	0.917	(0.882, 0.955)	<0.001	100
CTC spending on child education as mediator				
CTC spent on childcare				
Direct	0.926	(0.89, 0.965)	<0.001	87
Indirect	0.988	(0.981, 0.995)	0.001	13
Total	0.915	(0.88, 0.953)	<0.001	100
CTC spent on schoolbooks and supplies				
Direct	0.926	(0.888, 0.967)	<0.001	90
Indirect	0.991	(0.974, 1.009)	0.304	10
Total	0.918	(0.882, 0.955)	<0.001	100
CTC spent on school tuition				
Direct	0.920	(0.884, 0.958)	<0.001	93
Indirect	0.994	(0.989, 0.999)	0.027	7
Total	0.914	(0.879, 0.952)	<0.001	100
CTC spent on tutoring services				
Direct	0.916	(0.881, 0.954)	<0.001	98
Indirect	0.998	(0.997, 1.001)	0.065	2
Total	0.915	(0.879, 0.952)	<0.001	100
CTC spent on afterschool programs				
Direct	0.917	(0.881, 0.955)	<0.001	97
Indirect	0.997	(0.995, 1.001)	0.059	3
Total	0.914	(0.879, 0.952)	<0.001	100
CTC spent on transportation for school				
Direct	0.919	(0.884, 0.957)	<0.001	96
Indirect	0.996	(0.994, 0.999)	0.004	4
Total	0.916	(0.881, 0.954)	<0.001	100
CTC spent on recreational goods				
Direct	0.915	(0.879, 0.952)	<0.001	99
Indirect	0.999	(0.998, 1.001)	0.244	1
Total	0.914	(0.879, 0.952)	<0.001	100
CTC spending on household expenditure as mediator				
CTC spent on utilities and telecommunications				
Direct	0.948	(0.91, 0.988)	0.010	64
Indirect	0.969	(0.959, 0.98)	<0.001	36
Total	0.918	(0.883, 0.956)	<0.001	100
CTC spent on vehicle payments				
Direct	0.928	(0.892, 0.966)	<0.001	87
Indirect	0.988	(0.983, 0.994)	<0.001	13
Total	0.917	(0.882, 0.955)	<0.001	100
CTC spent on paying off credit cards or debts				
Direct	0.937	(0.899, 0.978)	0.002	73
Indirect	0.975	(0.964, 0.988)	<0.001	27
Total	0.914	(0.879, 0.952)	<0.001	100
CTC spent on savings or investments				
Direct	0.871	(0.836, 0.909)	<0.001	140
Indirect	1.043	(1.026, 1.06)	<0.001	−40
Total	0.908	(0.873, 0.946)	<0.001	100
CTC spent on charitable donations or giving to family				
Direct	0.914	(0.879, 0.952)	<0.001	100
Indirect	1.000	(1, 1.001)	0.947	0
Total	0.914	(0.879, 0.952)	<0.001	100

Note: Full estimation results are available upon request. The unweighted sample size was n = 773,186. CTC = Child Tax Credit. OR = odds ratio. CI = confidence interval.

**Table 3:** Results from mediation analysis on CTC spending patterns as mediators between CTC receipt and depression.

Mediator Variables	CTC Receipt–Anxiety Relationship			
	OR	(95% CI)	*p*	% of Total Effect
CTC spending on basic necessities as mediator				
CTC spent on food				
Direct	0.965	(0.918, 1.016)	0.170	47
Indirect	0.959	(0.936, 0.983)	0.001	53
Total	0.926	(0.885, 0.969)	0.001	100
CTC spent on rent or mortgage				
Direct	0.982	(0.938, 1.029)	0.437	30
Indirect	0.958	(0.943, 0.974)	<0.001	70
Total	0.940	(0.899, 0.984)	0.008	100
CTC spent on clothing				
Direct	0.929	(0.888, 0.973)	0.002	91
Indirect	0.992	(0.98, 1.005)	0.233	9
Total	0.922	(0.882, 0.965)	<0.001	100
CTC spending on child education as mediator				
CTC spent on childcare				
Direct	0.931	(0.89, 0.975)	0.002	87
Indirect	0.989	(0.981, 0.997)	0.006	13
Total	0.921	(0.881, 0.964)	<0.001	100
CTC spent on schoolbooks and supplies				
Direct	0.938	(0.894, 0.985)	0.010	81
Indirect	0.984	(0.965, 1.005)	0.125	19
Total	0.924	(0.884, 0.967)	0.001	100
CTC spent on school tuition				
Direct	0.924	(0.883, 0.967)	0.001	95
Indirect	0.996	(0.991, 1.002)	0.184	5
Total	0.920	(0.88, 0.963)	<0.001	100
CTC spent on tutoring services				
Direct	0.921	(0.881, 0.964)	<0.001	98
Indirect	0.999	(0.998, 1.001)	0.116	2
Total	0.920	(0.88, 0.963)	<0.001	100
CTC spent on afterschool programs				
Direct	0.922	(0.882, 0.965)	<0.001	97
Indirect	0.998	(0.995, 1.001)	0.150	3
Total	0.920	(0.88, 0.963)	<0.001	100
CTC spent on transportation for school				
Direct	0.926	(0.886, 0.969)	0.001	96
Indirect	0.997	(0.995, 1)	0.017	4
Total	0.923	(0.883, 0.966)	0.001	100
CTC spent on recreational goods				
Direct	0.920	(0.88, 0.963)	<0.001	100
Indirect	1.000	(0.999, 1.002)	0.852	0
Total	0.920	(0.88, 0.962)	<0.001	100
CTC spending on household expenditure as mediator				
CTC spent on utilities and telecommunications				
Direct	0.964	(0.921, 1.009)	0.114	54
Indirect	0.968	(0.957, 0.98)	<0.001	46
Total	0.933	(0.892, 0.976)	0.003	100
CTC spent on vehicle payments				
Direct	0.937	(0.896, 0.98)	0.004	87
Indirect	0.990	(0.984, 0.997)	0.002	13
Total	0.927	(0.887, 0.97)	0.001	100
CTC spent on paying off credit cards or debts				
Direct	0.944	(0.901, 0.99)	0.016	71
Indirect	0.976	(0.963, 0.989)	<0.001	29
Total	0.921	(0.881, 0.964)	<0.001	100
CTC spent on savings or investments				
Direct	0.896	(0.854, 0.942)	<0.001	133
Indirect	1.029	(1.008, 1.051)	0.007	−33
Total	0.922	(0.882, 0.965)	<0.001	100
CTC spent on charitable donations or giving to family				
Direct	0.920	(0.88, 0.963)	<0.001	100
Indirect	1.000	(1, 1.001)	0.638	0
Total	0.920	(0.88, 0.963)	<0.001	100

Note: Full estimation results are available upon request. The unweighted sample size was n = 773,186. CTC = Child Tax Credit. OR = odds ratio. CI = confidence interval.

## Data Availability

Not applicable.

## Supplementary Materials

The following supporting information can be downloaded at: https://www.mdpi.com/article/10.3390/ijerph20054425/s1, Supplementary Table S1: Sample Size by Survey Week and Phase; Supplementary Table S2: Household Pulse Survey Questions and Answer Choices for Variables in the Model; Supplementary Table S3: Pearson Correlation Coefficients (r) Between Mediating Variables; Supplementary Table S4: Descriptive Statistics of Samples across States.
