# Supplementary material for: Child Tax Credit, Spending Patterns, and Mental Health: Mediation Analyses of Data from the U.S. Census Bureau’s Household Pulse Survey during COVID-19
Source: Int J Environ Res Public Health. 2023 Mar 1;20(5):4425. doi: 10.3390/ijerph20054425 (PMC10002275; doi:10.3390/ijerph20054425)
Supplement: Supplementary file 1 [file ijerph-20-04425-s001.zip › ijerph-2180239-supplementary - final.pdf]

Supplementary Materials

This material includes supplementary tabulations that were referred in the main text.

Supplementary Table S1. Sample Size by Survey Week and Phase.

Survey Phase	Survey Week	Survey Dates	Count of Responses			
			(a) 1st-time	(b) 2nd-time	(c) 3rd-time	(a + b + c) Total
Phase 1	1	April 23–May 5, 2020	74,413	0	0	74,413
	2	May 7–12	22,411	19,585	0	41,996
	3	May 14–19	113,171	9,327	10,463	132,961
	4	May 21–26	69,184	28,318	3,713	101,215
	5	May 28–2	76,136	17,295	11,635	105,066
	6	June 4–9	60,079	16,383	6,840	83,302
	7	June 11–16	59,065	9,762	4,645	73,472
	8	June 18–23	92,661	11,986	3,415	108,062
	9	June 25–30	74,700	19,255	4,708	98,663
	10	July 2–7	68,281	15,315	7,171	90,767
	11	July 9–14	72,021	14,066	5,518	91,605
	12	July 16–21	67,896	14,130	4,766	86,792
Phase 2	13	August 19–31	109,051	0	0	109,051
	14	September 2–14	110,019	0	0	110,019
	15	September 16–28	99,302	0	0	99,302
	16	September 30–October 12	95,604	0	0	95,604
	17	October 14–26	88,716	0	0	88,716
Phase 3	18	October 28–November 9	58,729	0	0	58,729
	19	November 11–23	71,939	0	0	71,939
	20	November 25–December 7	72,484	0	0	72,484
	21	December 9–21, 2020	69,944	0	0	69,944
	22	January 6–18, 2021	68,348	0	0	68,348
	23	January 20–February 1	80,567	0	0	80,567
	24	February 3–15	77,122	0	0	77,122
	25	February 17–March 1	77,788	0	0	77,788
	26	March 3–15	78,306	0	0	78,306
	27	March 17–29	77,104	0	0	77,104
Phase 3.1	28	April 14–26	68,913	0	0	68,913
	29	April 28–May 10	78,467	0	0	78,467
	30	May 12–24	72,897	0	0	72,897
	31	May 26–June 7	70,854	0	0	70,854
	32	June 9–21	68,067	0	0	68,067
	33	June 23–July 5	66,262	0	0	66,262
Phase 3.2	34	July 21–August 2	64,562	0	0	64,562
	35	August 4–16	68,799	0	0	68,799
	36	August 18–30	69,114	0	0	69,114
	37	September 1–13	63,536	0	0	63,536
	38	September 15–27	59,833	0	0	59,833

	39	September 29–October 11	57,064	0	0	57,064
Phase 3.3	40	December 1–13	60,826	0	0	60,826
	41	December 29, 2021–January 10, 2022	74,995	0	0	74,995
	42	January 26–February 7	75,482	0	0	75,482
Phase 3.4	43	March 2–14	84,158	0	0	84,158
	44	March 30–April 11	63,769	0	0	63,769
	45	April 27–May 9	61,767	0	0	61,767
Phase 3.5	46	June 1–13	62,826	0	0	62,826
	47	June 29–July 11	58,304	0	0	58,304
	48	July 27–August 8, 2022	46,801	0	0	46,801
		Sum of Weeks 1 to 48	3,482,337	175,422	62,874	3,720,633
		% of Total Responses	93.6	4.7	1.7	100.0

Notes: The shaded rows present the period of analysis. This table is an updated and revised version of Supplementary Table 1 in Park & Kim (2021), presenting the unweighted count of responses. If weighted by person-weight (*pweight* in HPS microdata), each survey week represents the adult (ages 18+) population of 249,170,916 in 2020, 250,265,449 in 2021, and 252,481,011 in 2022. If weighted by household-weight (*hweight* in HPS microdata; only available in phase 2 and later phases of HPS), each week represents the total households of 121,520,180 in 2020, 122,798,192 in 2021, and 124,345,410 in 2022.
Sources: U.S. Census Bureau's Household Pulse Survey (HPS) Public Use File (PUF).

Supplementary Table S2. Household Pulse Survey Questions and Answer Choices for Variables in the Model.

Variable	Survey Question	Survey Answer Choices
**Exposures**		
Receipt of CTC during the COVID-19 pandemic	In the last 4 weeks, did you receive a refund from your 2021 tax return?	1) Yes, 2) No
	Thinking about your use of the payments from the "Child Tax Credit" did you:	1) Mostly spend it<br>2) Mostly save it<br>3) Mostly use it to pay off debt
**Mediators**		
CTC spending on basic necessities		
Food	What did you and your household mostly spend the "Child Tax Credit" portion of your refund on? Select all that apply. Food (groceries, eating out, take out)	1) Yes, 2) No
Rent or mortgage	Rent or mortgage (scheduled or monthly)	1) Yes, 2) No
Clothing	Clothing (clothing, accessories, shoes)	1) Yes, 2) No
CTC spending on child education		
Childcare	Childcare (formal facility, paying family or caregiver directly)	1) Yes, 2) No
Schoolbooks and supplies	School books and supplies	1) Yes, 2) No
School tuition	School tuition	1) Yes, 2) No
Tutoring services	Tutoring services	1) Yes, 2) No
Afterschool programs	After school programs (other than tutoring and childcare)	1) Yes, 2) No
Transportation for school	Transportation for school (bus service, metro, etc..)	1) Yes, 2) No
Recreational goods	Recreational goods (sports and fitness equipment, bicycles, toys, games)	1) Yes, 2) No
CTC spending on household expenditure		
Utilities and telecommunications	Utilities and telecommunications (natural gas, electricity, cable, internet, cellphone)	1) Yes, 2) No
Vehicle payments	Vehicle payments (scheduled or monthly)	1) Yes, 2) No
Paying down credit card or debts	Paying down credit card, student loans, or other debts	1) Yes, 2) No
Savings or investments	Savings or investments	1) Yes, 2) No
Charitable donations or giving to family	Charitable donations or giving to family members	1) Yes, 2) No
**Covariates**		
Demographic characteristics		
Age	What year were you born? Please enter a number.	1932–2002
Gender	Are you?	1) Male<br>2) Female

Race/ethnicity	Are you of Hispanic, Latino, or Spanish origin? - Selected Choice	1) No, not of Hispanic, Latino, or Spanish origin<br>2) Yes, of Hispanic, Latino, or Spanish origin
	What is your race? Please select all that apply. - Selected Choice	1) White, Alone<br>2) Black, Alone<br>3) Asian, Alone<br>4) Any other race alone, or race in combination
Marital status	What is your marital status?	1) Now married<br>2) Widowed<br>3) Divorced<br>4) Separated<br>5) Never married
Children in household	How many people under 18 years-old currently live in your household? Please enter a number.	(0–40) number of people under 18 (whole number)
Household size	How many total people-adults and children-currently live in your household, including yourself? Please enter a number.	(1–40) number of people (whole number)
Socioeconomic status (SES)		
Education	What is the highest degree or level of school you have completed?	1) Less than high school<br>2) Some high school<br>3) High school graduate or equivalent (for example GED)<br>4) Some college, but degree not received or is in progress<br>5) Associate's degree (for example AA, AS)<br>6) Bachelor's degree (for example BA, BS, AB)<br>7) Graduate degree (for example master's, professional, doctorate)
Household income	In 2019 what was your total household income before taxes?	1) Less than $25,000<br>2) $25,000–$34,999<br>3) $35,000–$49,999<br>4) $50,000–$74,999<br>5) $75,000–$99,999<br>6) $100,000–$149,999<br>7) $150,000–$199,999<br>8) $200,000 and above

Health insurance status		
Public health insurance	Are you currently covered by any of the following types of health insurance or health coverage plans? Mark Yes or No for each.	1) Yes, Has Public Health Insurance<br>2) No Public Health Insurance
Private health insurance	Are you currently covered by any of the following types of health insurance or health coverage plans? Mark Yes or No for each.	1) Yes, Has Private Health Insurance<br>2) No Private Health Insurance
Location of residence		
15 largest metropolitan statistical area	Data based on the HPS respondent's complete current street address	–
States and Washington, D.C.	Data based on the HPS respondent's complete current street address	–

Sources: U.S. Census Bureau’s Household Pulse Survey (HPS) Public Use File (PUF).

Supplementary Table S3. Pearson Correlation Coefficient (*r*) Between Mediating Variables.

	Food	Rent	Clothing	Child care	School books	School tuition	Tutoring	After school	Transport	Recreation	Utilities	Vehicle	Credit card	Savings	Donations
Food	1.000														
Rent	0.319	1.000													
Clothing	0.426	0.186	1.000												
Childcare	0.086	0.047	0.052	1.000											
School books	0.218	0.095	0.376	0.016	1.000										
School tuition	0.032	0.021	0.042	0.070	0.128	1.000									
Tutoring	0.042	0.014	0.052	0.039	0.096	0.083	1.000								
After school	0.080	0.032	0.100	0.115	0.118	0.072	0.108	1.000							
Transport	0.137	0.139	0.159	0.043	0.141	0.050	0.056	0.076	1.000						
Recreation	0.106	0.062	0.185	0.015	0.117	0.032	0.052	0.130	0.089	1.000					
Utilities	0.367	0.508	0.240	0.011	0.132	0.004	0.008	0.025	0.164	0.080	1.000				
Vehicle	0.212	0.386	0.152	0.044	0.084	0.024	0.019	0.038	0.114	0.074	0.393	1.000			
Credit card	0.066	0.161	0.039	−0.008	0.022	0.007	0.011	0.011	0.036	0.041	0.180	0.233	1.000		
Savings	−0.281	−0.164	−0.155	−0.099	−0.114	−0.055	−0.025	−0.045	−0.057	−0.020	−0.187	−0.099	−0.130	1.000	
Donations	0.036	0.049	0.050	0.008	0.036	0.032	0.045	0.038	0.036	0.105	0.045	0.049	0.050	0.046	1.000

Note. This table presents Pearson correlation coefficient (*r*) between mediating variables in the model. *Source.* U.S. Census Bureau's Household Pulse Survey Public Use File (PUF).

Supplementary Table S4. Descriptive Statistics of Samples across States in the U.S.

Variables	Full Sample (n = 98,026), %, Mean (SD)	Generalized Anxiety Disorder		Major Depressive Disorder	
		Yes (n = 30,604), %, Mean (SD)	No (n = 67,422), %, Mean (SD)	Yes (n = 20,367), %, Mean (SD)	No (n = 77,659), %, Mean (SD)
Location of residence					
States and Washington, D.C.					
Alabama	1.6	1.7	1.5	1.8	1.5
Alaska	0.2	0.2	0.2	0.2	0.3
Arizona	2.5	2.6	2.5	2.6	2.5
Arkansas	1.0	1.2	0.9	1.3	0.9
California	10.8	10.2	11.2	10.4	11.0
Colorado	1.8	1.6	1.8	1.6	1.8
Connecticut	1.0	1.0	1.0	0.9	1.0
Delaware	0.3	0.3	0.3	0.3	0.3
District of Columbia	0.1	0.1	0.2	0.1	0.1
Florida	6.1	7.2	5.6	6.8	5.9
Georgia	3.4	3.4	3.4	3.4	3.4
Hawaii	0.5	0.4	0.5	0.5	0.5
Idaho	0.7	0.6	0.7	0.6	0.7
Illinois	3.6	3.2	3.8	3.2	3.7
Indiana	2.1	2.1	2.2	2.1	2.2
Iowa	1.0	0.9	1.1	0.8	1.1
Kansas	0.9	0.9	1.0	0.9	1.0
Kentucky	1.5	1.6	1.4	1.7	1.4
Louisiana	1.3	1.7	1.2	1.8	1.2
Maine	0.4	0.5	0.4	0.4	0.4
Maryland	1.9	1.8	2.0	1.9	2.0
Massachusetts	1.9	1.7	2.0	1.6	2.0
Michigan	2.9	2.9	3.0	2.9	2.9
Minnesota	1.7	1.4	1.8	1.3	1.9
Mississippi	1.0	1.1	0.9	1.1	0.9
Missouri	1.9	2.0	1.9	2.1	1.9
Montana	0.3	0.3	0.3	0.3	0.3
Nebraska	0.6	0.5	0.7	0.5	0.7
Nevada	1.0	1.1	0.9	1.3	0.9
New Hampshire	0.4	0.4	0.4	0.4	0.4
New Jersey	2.5	2.2	2.6	2.2	2.5
New Mexico	0.7	0.7	0.7	0.7	0.7
New York	5.4	4.8	5.7	4.6	5.6
North Carolina	3.3	3.3	3.4	3.0	3.4
North Dakota	0.3	0.2	0.3	0.2	0.3
Ohio	3.8	3.8	3.8	3.7	3.9
Oklahoma	1.3	1.5	1.3	1.6	1.3
Oregon	1.3	1.4	1.3	1.3	1.3
Pennsylvania	3.6	3.7	3.5	3.7	3.5
Rhode Island	0.3	0.3	0.3	0.2	0.3
South Carolina	1.7	1.5	1.8	1.5	1.8
South Dakota	0.3	0.3	0.3	0.3	0.3
Tennessee	2.1	2.4	2.0	2.6	2.0
Texas	9.8	10.3	9.5	10.7	9.5
Utah	1.2	1.2	1.3	1.2	1.2
Vermont	0.2	0.2	0.2	0.2	0.2
Virginia	2.8	2.7	2.8	2.8	2.8
Washington	2.3	2.5	2.3	2.4	2.3

West Virginia	0.6	0.6	0.5	0.7	0.5
Wisconsin	1.7	1.4	1.9	1.5	1.8
Wyoming	0.2	0.2	0.2	0.2	0.2

Note. This table supplements Table 1 in main text.
